# Aprepitant for the Treatment of Chronic Refractory Pruritus

**DOI:** 10.1155/2017/4790810

**Published:** 2017-09-19

**Authors:** Alice He, Jihad M. Alhariri, Ronald J. Sweren, Madan M. Kwatra, Shawn G. Kwatra

**Affiliations:** ^1^Department of Dermatology, Johns Hopkins University School of Medicine, Baltimore, MD, USA; ^2^Department of Anesthesiology, Duke University Medical Center, Durham, NC, USA

## Abstract

Chronic pruritus is a difficult condition to treat and is associated with several comorbidities, including insomnia, depression, and decreased quality of life. Treatment for chronic itch includes corticosteroids, antihistamines, and systemic therapies such as naltrexone, gabapentin, UV light therapy, and immunomodulatory treatments, including azathioprine, methotrexate, and cellcept. However, some patients still remain refractory to conventional therapy. Aprepitant is a neurokinin-1 receptor antagonist approved for the prevention of chemotherapy-induced and postoperative nausea and vomiting (CINV, PONV). Recently, aprepitant has demonstrated effectiveness in several case series and open label trials in relieving pruritus for patients refractory to other treatments. Patients with pruritus associated with Sézary syndrome, mycosis fungoides, lung adenocarcinoma, breast carcinoma, sarcomas, metastatic solid tumors, chronic kidney disease, hyperuricemia, iron deficiency, brachioradial pruritus, and Hodgkin's lymphoma have experienced considerable symptom relief with short-term use of aprepitant (up to two weeks). Due to differences in reporting and evaluation of drug effects, the mechanism of aprepitant's role is difficult to understand based on the current literature. Herein, we evaluate aprepitant's antipruritic effects and discuss its mechanism of action and adverse effects. We propose that aprepitant is an alternative for patients suffering from pruritus who do not obtain enough symptom relief from conventional therapy.

## 1. Introduction

Chronic itch is a distressing condition for patients with a significant effect on quality of life. If a patient is nonresponsive to topical therapy, there are limited systemic options available. Current options include corticosteroids, antihistamines, capsaicin, naltrexone, gabapentin, UV light therapy, and immunomodulatory treatments such as azathioprine, methotrexate, and cellcept. The purpose of this review is to make dermatologists aware of aprepitant as a medication that is effective for treating subsets of patients with chronic refractory pruritus.

Aprepitant was first approved in the United States in March 2003 to prevent chemotherapy-induced nausea and vomiting (CINV) [1]. The drug was originally developed to treat depression, but clinical trials failed to demonstrate an effect in a nontoxic dosage range [2]. Aprepitant is a neurokinin-1 (NK1) receptor antagonist and is the first of its class to be approved for use [3]. Aprepitant exerts its effects by blocking substance P (SP), an endogenous ligand of the NK1 receptor. Substance P mediates several physiologic processes, including pain, depression, nausea, vomiting, and pruritus [3]. As such, there is much excitement over the potential for developing NK1 receptor antagonists as a therapy for many disease states.

Recently clinicians have discovered an off-label use for aprepitant to treat chronic refractory pruritus. Concerns for aprepitant use include its high cost and potential interactions with multiple other drugs. Herein we review aprepitant's efficacy as an antipruritic agent, mechanisms of action, and adverse effects.

## 2. Materials and Methods

In December 2015 through April 2017, we conducted a literature search of PubMed, Ovid MEDLINE, clinicaltrials.gov, and Google Scholar for key word combinations of “aprepitant” coupled with “pruritus,” “itch,” and “antipruritic.” All results were checked for relevance.

## 3. Results and Discussion

Our search yielded a total of 143 results (with redundancy) from 2001 to 2017 containing the aforementioned key words. Ultimately, 16 clinical articles were included in this review as they focused specifically on chronic refractory pruritus in humans. Only reports published in English were included.

## 4. Clinical Antipruritic Therapy of Aprepitant

There have been several case series and open label trials reported in literature about the efficacy of aprepitant to treat refractory chronic pruritus. A total of 73 patients were included in the reports reviewed here [4–18].

### 4.1. Pruritus Associated with Malignant Conditions

The first reported use of aprepitant for refractory pruritus was by Duval and Dubertret in 2009 [4]. Duval and Dubertret reported on three patients with Sézary syndrome who were all hospitalized for pruritus refractory to conventional therapy [4]. The visual analogue scale (VAS) score was used to assess pruritus severity, with 0 indicating no pruritus and 10 representing the worst possible case of pruritus imaginable. Quality of life was assessed using the Dermatology Life Quality Index (DLQI) questionnaire out of 30, with higher scores representing worse quality of life. After just one dose of 80 mg aprepitant, average VAS score for itch dropped from 8 to 2.33, and after one week the mean DLQI score improved from 19.5 to 6 [4]. All patients reported diminished insomnia and better quality of sleep [4]. However, the treatment had no effect on erythroderma [4]. The ability of aprepitant to treat refractory chronic pruritus associated with cutaneous T-cell lymphomas was further confirmed by several subsequent case reports in a total of 17 patients (nine with Sézary syndrome, seven with mycosis fungoides, and one with cutaneous anaplastic large cell lymphoma) [9, 10, 12, 14, 18]. Aprepitant regimens for these patients included either a 3-day course of 125 mg/80 mg/80 mg repeated every two weeks or daily 80 mg aprepitant. All patients showed symptom improvement within as early as three hours to two weeks, except for one patient who failed to respond to aprepitant at all. Average VAS score dropped from 9.53 to 3.03, and average DLQI score improved from 22.57 to 8. In this cohort, only one patient experienced a self-limited headache on the first day of aprepitant therapy [14], and one patient relapsed with substantial worsening of pruritus on the 12th day of treatment after an initial good response to aprepitant [16], but this patient's reaction was believed to be due to underlying disease progression; no other adverse reactions were reported.

Aprepitant has also been reported to treat chronic pruritus associated with solid tumors. Several case series have reported on a total of 29 patients with a variety of solid tumors, including lung adenocarcinoma, breast carcinoma, and soft tissue sarcomas [6–8, 11]. Most patients underwent anticancer treatment with erlotinib [6, 8, 11]. All patients failed conventional therapy for pruritus and were given aprepitant regimens of 125 mg/80 mg/80 mg for three consecutive days, 125 mg/80 mg/80 mg every other day for five days, or 80 mg daily. Average VAS scores dropped from 6.96 to 0.93, before and after aprepitant therapy. No adverse reactions were reported for any patients.

### 4.2. Pruritus Associated with a Variety of Nonmalignant Conditions

In 2010, Ständer et al. treated a cohort of 20 patients with chronic refractory pruritus associated with chronic kidney disease, hyperuricemia, and iron deficiency [5]. Majority of the patients also had severe prurigo nodularis. Patients were given 80 mg aprepitant daily for 3–13 days (mean 6.6 days). Mean VAS score improved from 8.4 to 4.9 for all patients, which included four patients who did not respond to aprepitant therapy at all. Interestingly, the authors found that patients with atopic diathesis and/or prurigo nodularis experienced greater reduction of pruritus (mean VAS score reduction of 50.9%) with clinical improvement of scratch lesions, as compared to patients without dermatological diseases (mean VAS score reduction of 26.3%). Three patients experienced adverse reactions of nausea, vertigo, and drowsiness, but none required cessation of aprepitant therapy.

One case in literature reports aprepitant's efficacy in treating refractory brachioradial pruritus [13]. The patient experienced vast improvement of pruritus and scratch lesions with just two days of 80 mg aprepitant daily for seven days. However, relapse occurred just 48 hours after the last dose of aprepitant. In contrast, aprepitant's antipruritic effects lasted for six weeks in a report of a man with pruritus of unknown origin with superficial psoriasiform dermatitis treated with 125 mg/80 mg/80 mg/80 mg aprepitant for 4 days [17]. He experienced significant symptom relief (VAS score 8 to 1 and DLQI 24 to 8, before and after treatment) with no adverse effects [17]. There is one report on treating chronic pruritus associated with Hodgkin's lymphoma with aprepitant [15]. Treatment with 80 mg aprepitant daily for two weeks dropped the patient's VAS score from 9 to 5 and allowed the patient to lead a better quality of life. Finally, one case reports on aprepitant treating refractory pruritus of unclear origin [17]. An aprepitant regimen of 125 mg on day 1 and then 80 mg on days 2–4 resulted in VAS score improvement of 8/10 to 4/10 after 24 hours, and it improved to 1/10 after six weeks [17]. The patient experienced no adverse effects and greatly improved insomnia and cutaneous lesions [17].

### 4.3. Summary of Clinical Effects

In summary, a total of 73 patients were included in this review who were treated with aprepitant for chronic pruritus associated with cutaneous T-cell lymphomas (27%), solid tumors (40%), and a variety of other conditions (33%). Several patients suffered from a decreased quality of life due to pruritus-related side effects, including insomnia (8 reported patients) and scratch lesions (27 reported patients). All patients previously failed conventional therapy, often consisting of oral antihistamines and topical steroids. Aprepitant treatment regimens varied by underlying disease (see Table 1). Initial mean VAS score for 71 patients was 8.1 (VAS score not reported for 2 patients). After initiation of aprepitant therapy, mean VAS score improved to 2.7. Time to improvement ranged from three hours to two weeks. Nearly all patients experienced symptomatic relief from pruritus with aprepitant, including reduction of scratch lesions (63% of those reported) and reduced insomnia. Four patients (5.6%) did not respond to aprepitant therapy at all. Mild adverse reactions were reported in only four patients (5.6%), and one patient experienced substantial worsening of pruritus during treatment after an initial good response due to underlying disease progression and required cessation of aprepitant. No patients required cessation of aprepitant therapy due to drug-related adverse effects. Relapse trends were variable; nine patients were reported to have pruritus symptom relapse 48–72 hours after last aprepitant dose, while six patients were reported to have stable symptom control over two weeks after last aprepitant dose. Five patients continued with aprepitant prophylaxis at last follow-up with good symptom control.

The shared conclusion from the reviewed reports is that SP is indeed an important mediator of pruritus in many diseases and that aprepitant exhibits antipruritic activity as a NK1 receptor and SP antagonist. The authors agree that there is enough convincing evidence to warrant multicenter randomized, controlled, clinical trials to truly assess the efficacy of aprepitant's antipruritic effect. Additional trials will be necessary not only to delineate the optimal dosage and therapeutic interval for aprepitant's antipruritic effects, but also to understand the pruritic disease states that will benefit most from aprepitant, as pruritic pathomechanisms differ among various underlying diseases.

## 5. Pharmacology

Aprepitant is a selective, high-affinity antagonist of NK1 receptors, with little to no affinity for serotonin, dopamine, and corticosteroid receptors [19]. The oral bioavailability of aprepitant is approximately 60–65% at the recommended dose range of 80–125 mg and achieves peak plasma concentrations (*T*_max_) in 4 hours [3, 19]. The mean apparent volume of distribution at steady-state is 66 L in humans, where the drug is highly bound to plasma proteins (>95%) [3]. Aprepitant is able to cross the blood-brain barrier in humans into the central nervous system (CNS) [3]. Aprepitant undergoes extensive metabolism in the body and is eliminated primarily by metabolism; it is not renally excreted [19]. Aprepitant is metabolized largely by cytochrome P450 3A4 isoform (CYP3A4), which occurs mainly by oxidation at its morpholine ring and its side chains [19]. Aprepitant has a plasma clearance of approximately 62–90 ml/min, and its terminal half-life ranges from 9 to 13 hours [19].

## 6. Side Effects

Aprepitant does not have acute adverse events [19]. The side effects of aprepitant therapy have been monitored in clinical trials in patients using aprepitant for depression and prevention of CINV and PONV.

Kramer et al. and Keller et al. reported on a total of 2739 patients taking up to 300 mg oral aprepitant for up to eight weeks for depression. Both reports found no significant differences in adverse events between aprepitant versus placebo and concluded that aprepitant is generally well tolerated over a long period (up to eight weeks) [1, 20, 21].

In two active-controlled, double-blind, clinical trials that compared aprepitant in combination with ondansetron and dexamethasone (aprepitant regimen; *n* = 1412) to ondansetron and dexamethasone alone (standard therapy; *n* = 1396) for prevention of CINV, the most common adverse reactions that occurred in >3% of adults in both regimens included fatigue, diarrhea, asthenia, dyspepsia, abdominal pain, hiccups, decreased white blood cell count, dehydration, constipation, hypotension, and increased alanine aminotransferase (see Table 2) [19]. The type of adverse reactions was similar for patients in both groups, but the incidence of each adverse reaction was consistently 1-2% higher in the aprepitant regimen group as compared to those on standard therapy [19].

Two active-controlled, double-blind, clinical studies compared 40 mg oral aprepitant (*N* = 564) to 4 mg intravenous ondansetron (*N* = 538) for the prevention of PONV [19]. Common adverse reactions experienced by >3% of adults included constipation and hypotension, with a 1% higher incidence of both in those treated with aprepitant as compared to ondansetron [19].

Aprepitant is approved for use in children > 12 years of age or in children < 12 years who weigh 30 kg [19]. Two active-controlled clinical trials in pediatric patients compared aprepitant and ondansetron (aprepitant regimen; *n* = 184) to ondansetron with or without dexamethasone (control regimen; *n* = 168) for CINV [19]. Common adverse events experienced in >3% of the pediatric population for the prevention of CINV include neutropenia, headache, diarrhea, decreased appetite, cough, fatigue, decreased hemoglobin, dizziness, and hiccups [19]. The incidence of each adverse event was consistently 1–4% higher in the aprepitant regimen than in the control regimen [19].

## 7. Contraindications and Warnings

There are two strict contraindications to aprepitant therapy according to aprepitant's pharmaceutical drug label package: (1) known hypersensitivity to any component of the drug and (2) concurrent use with pimozide [19]. Aprepitant is a substrate, moderate inhibitor, and inducer of CYP3A4 [19]. As such, there are risks of many drug-drug pharmacokinetic interactions. Pimozide is a CYP3A4 substrate, thereby inhibition of CYP3A4 by aprepitant could increase plasma levels of pimozide, increasing risk of serious adverse reactions such as QT prolongation, a known adverse effect of pimozide [19].

Coadministration of other CYP3A4 substrates with aprepitant also requires caution and careful monitoring. Concurrent use of aprepitant with warfarin yields a risk of decreased INR [19]. Likewise, efficacy of hormonal contraceptives may be reduced with concurrent use with aprepitant and up to 28 days after the last dose of aprepitant [19]. Erlotinib is a commonly used anticancer therapy that is primarily metabolized and cleared by CYP3A4 [22]. Aprepitant has been shown to significantly decrease erlotinib clearance and increase its plasma concentration [8]. Strict monitoring and surveillance of drug plasma concentrations are necessary when administering aprepitant with erlotinib and other chemotherapy agents metabolized by CYP3A4.

Since aprepitant is also a CYP3A4 substrate, its plasma concentration needs to be carefully monitored when coadministered with other CYP3A4 inhibitors, which may increase risk of adverse reactions, or inducers, which may reduce the drug's efficacy (see Table 3) [19].

## 8. Mechanism of the Antipruritic Effect

The main antipruritic effect of aprepitant is via substance P (SP) antagonism. SP plays a major role in the induction and maintenance of pruritus in the skin [1, 23–26]. SP is a short neuropeptide that preferentially binds to the NK1 receptor expressed in the CNS and on immune cells, cutaneous keratinocytes, and mast cells [27]. An increase in NK1 receptor expression has been reported on keratinocytes in pruritic skin conditions [28, 29]. Upon binding to its receptor on keratinocytes, fibroblasts, and mast cells in the skin, SP stimulates the secretion of a variety of inflammatory factors, including interferon-*γ*, IL-1*β*, IL-8, histamine, leukotriene B4, prostaglandin D2, and vascular endothelial growth factor (VEGF) [27]. This leads to vasodilation of vessels and neurogenic inflammation, which clinically presents as erythema, edema, and pruritus [27].

Aprepitant is a highly selective NK1 receptor antagonist with little to no affinity for other neurokinin receptors [10, 11]. By blocking mast cell degranulation, aprepitant is able to inhibit SP-mediated inflammation and pruritus [28, 30]. This conclusion is further supported by reports that aprepitant is more effective at relieving pruritus in patients with prurigo nodularis, as prior studies have found that patients with prurigo nodularis have an increased number of nerve fibers positive for SP [5, 31].

Another hypothesis is that oral aprepitant acts on the CNS [32]. Wallengren reported that pruritus failed to respond to local treatment with 5% topical aprepitant despite correct cutaneous absorption [32]. While this author agrees that aprepitant's antipruritic effect is via SP antagonism, she suggests that the effect is in the CNS and not the skin [32].

## 9. Conclusions

In the studies reviewed here, aprepitant has successfully treated chronic refractory pruritus associated with cutaneous T-cell lymphomas, solid tumors, chronic kidney disease, and Hodgkin's lymphoma, among others. These patients experienced considerable pruritic symptom relief and improvement in pruritus-induced comorbidities, such as insomnia, depression, and significant reductions in quality of life. Few patients experienced adverse effects. Collectively, these reports demonstrate the potential for oral aprepitant to be an alternative antipruritic treatment for patients who are insufficiently relieved by conventional therapy. However, these conclusions must be taken in the context of aprepitant's high cost and potential interaction with multiple other drugs [33]. The high cost of aprepitant prevents its wide use and more economical antipruritic therapies should be attempted first. Even though aprepitant has been shown in these case series to produce a dramatic improvement in pruritus symptoms, unfortunately it may have to be used as a last resort due to high economic barriers. Additional studies are needed to clarify the optimal dosage for aprepitant's antipruritic effects and to determine in which disease states aprepitant will be most effective and applicable.

## Figures and Tables

**Table 1:** Aprepitant dosing by pruritus-associated disease based on prior studies.

Sézary syndrome	80 mg daily for 10–15 days and then 80 mg on alternate days for 1.5–23 weeks [4, 10, 16] OR 125 mg/80 mg/80 mg for 3 days, in 2-week repetitions for 6–24 weeks [9]

Mycosis fungoides	80 mg daily for 4 months or longer for prophylaxis [12] OR 125 mg/80 mg/80 mg for 3 days, in 2-week repetitions for 6–24 weeks [9, 14]

Cutaneous anaplastic large cell lymphoma	125 mg/80 mg/80 mg for 3 days [18]

Lung adenocarcinoma	80 mg daily continuously for prophylaxis [8] OR 125 mg/80 mg/80 mg for 3 days, then alternating days of 125 mg and 80 mg for 2 months or longer for prophylaxis [11]

Breast carcinoma	Day 1: 125 mg, day 3: 80 mg, day 5: 80 mg [7]

Metastatic solid tumors	Day 1: 125 mg, day 3: 80 mg, day 5: 80 mg [11]

Soft tissue sarcoma	Day 1: 125 mg, day 3: 80 mg, day 5: 80 mg [7]

Hodgkin's lymphoma	80 mg daily for 2 weeks [15]

Chronic kidney disease	80 mg daily for 3–13 days [5]

Hyperuricemia	80 mg daily for 3–13 days [5]

Iron deficiency	80 mg daily for 3–13 days [5]

Pruritus of unclear origin	125 mg on day 1, 80 mg on days 2–4 [17]

Brachioradial pruritus	80 mg daily for 7 days; repeat if relapse [13]

**Table 2:** Aprepitant side effects reported in ≥3% of patients by organ system for adults and pediatric patients [5, 14, 19].

Adult population	
Neurologic	Fatigue, vertigo, drowsiness, headache
Gastrointestinal and hepatic	Diarrhea, constipation, dyspepsia, abdominal pain, increased alanine aminotransferase, nausea
Neuromuscular and musculoskeletal	Asthenia, hiccups
Hematologic	Decreased WBC count
Endocrine and metabolic	Dehydration
Cardiovascular	Hypotension

Pediatric population	

Neurologic	Headache, fatigue, dizziness
Gastrointestinal	Diarrhea, decreased appetite
Neuromuscular and musculoskeletal	Cough, hiccups
Hematologic	Neutropenia, decreased hemoglobin

**Table 3:** Drugs that may interact with aprepitant if used concurrently, based on CYP3A4 interactions.

CYP3A4 interaction	CYP3A4 substrates	CYP3A4 inducers	CYP3A4 inhibitors
Risk	Risk of increased or decreased plasma levels with concurrent aprepitant use	Risk of decreased aprepitant plasma levels	Risk of increased aprepitant plasma levels

Drugs	Pimozide	Rifampin	Ketoconazole
	Erlotinib		Diltiazem
	Warfarin		
	Hormonal contraceptives		
	Ifosfamide		
	Methylprednisolone		
	Dexamethasone		
